# Characterization of a Thermophilic and Acidophilic GH78 α-L-Rhamnosidase from *Thermotoga* sp. 2812B Capable of Efficiently Hydrolyzing a Variety of Natural Flavonoid Diglycosides

**DOI:** 10.3390/biom16010068

**Published:** 2025-12-31

**Authors:** Bin-Chun Li, Weijuan Dong, Bingbing Wu, Yanlong Liu, Na Han, Guo-Bin Ding

**Affiliations:** 1 Institute of Biotechnology, Key Laboratory of Chemical Biology and Molecular Engineering of Ministry of Education, Shanxi University, Taiyuan 030006, China; 2 Shanxi Key Laboratory of Biotechnology, Shanxi University, Taiyuan 030006, China; 3 School of Life Science, Shanxi University, Taiyuan 030006, China; 4 Institutes of Biomedical Sciences, Inner Mongolia University, Hohhot 010070, China

**Keywords:** α-L-Rhamnosidase, thermophilic, acidophilic, natural flavonoid glycosides, glycoside hydrolase family 78, flavonoid glucosides

## Abstract

α-L-Rhamnosidase can specifically hydrolyze plant natural glycosides and holds significant potential for biocatalytic applications in functional foods, healthy products, and pharmaceutical industries. Herein, a novel thermophilic and acidophilic α-L-rhamnosidase TsRha from *Thermotoga* sp. 2812B belonging to glycoside hydrolase family 78 was identified by genome mining and comprehensively characterized by bioinformatics, computer-aided structural analysis, and biochemical characterization. TsRha possesses a domain architecture comprising one catalytic (α/α)_6_-barrel domain and four β-sheet domains. TsRha displayed optimal activity at 90 °C and pH 5.0, remarkable thermostability at 80 °C, and considerable tolerance to organic solvents. TsRha exhibited broad substrate selectivity and might efficiently hydrolyze a series of natural flavonoid glycosides with various glycosidic bonds (α-1, α-1, 2, α-1, 6) from different aglycone subgroups (flavanone, flavone, flavonol, and dihydrochalcone). Moreover, it demonstrated high conversion efficiencies toward a variety of natural flavonoid diglycosides rutin, naringin, naringin dihydrochalcone, hesperidin, and troxerutin, achieving ≥99.1% conversion within 20~100 min. The excellent properties including high activity, thermophilicity, acidophilicity, good thermostability, broad substrate spectrum will make the α-L-rhamnosidase TsRha a promising biocatalyst for the efficient production of rare and high-value flavonoid glucosides with improved bioavailability and bioactivity.

## 1. Introduction

α-L-Rhamnosidase (EC 3.2.1.40) is widely distributed in nature, with primary sources including microorganisms, plants, and animals [1]. As a member of the glycoside hydrolase family, α-L-rhamnosidase specifically catalyzes the hydrolysis of α-L-rhamnose moiety from the non-reducing ends of various rhamnosides. Its functions encompass the depolymerization of pectin [2] and the de-rhamnosylation of plant natural glycosides (PNGs) such as natural flavonoid glycosides [3], steroid saponins [4], and ginsenosides [5]. Acting on the α-L-rhamnosyl groups of natural flavonoid glycosides, α-L-rhamnosidase cleaves α-1, 2, α-1, 3, α-1, 4, α-1, and α-1, 6 glycosidic bonds. Its role as a key biotechnological tool in the production of functional foods and pharmaceuticals has been well recognized. In food industry, α-L-rhamnosidase can enhance the flavor of citrus juices by hydrolyzing the bitter naringin [6]. Moreover, α-L-rhamnosidases can be applied to enzymatically modify natural flavonoid glycosides with various pharmacological effects [7]. This modification significantly improves their bioactivity [8,9], water solubility [10,11], and bioavailability [12]. Consequently, the biocatalytic preparation of rare and high-value flavonoid glucosides can be achieved through the enzymatic hydrolysis of cheap natural flavonoid glycosides using the α-L-rhamnosidases, such as the conversion of naringin to prunin [13], rutin to isoquercitrin [14], and notably the production of icaritin and its glycoside icariside I from icariin and epimedin C [14,15,16].

Based on amino acid sequence similarity, α-L-rhamnosidases are classified into the glycoside hydrolase (GH) families 28, 78, and 106. Among these, the GH78 α-L-rhamnosidases (Rha78s) have been the most extensively investigated, with the majority from microorganisms, particularly bacteria. To date, 49 microbial Rhs78s have been cloned and characterized according to the CAZy database [17]. Bacterial Rha78s predominate (32 in total), especially lactic acid bacteria and thermophilies including *Dictyoglomus thermophilum* H-6-12 [18], *Thermoclostridium stercorarium* NCIB 11754 [19], *Thermoclostridium stercorarium* subsp. thermolacticum DSM 2910 [20], *Thermomicrobia* bacterium PRI-1686 [21] and *Thermotoga petrophila* RKU-1 [22,23]. Characterization of various bacterial and fungal Rha78s have revealed considerable diversity in their enzymatic properties. Bacterial Rha78s generally exhibit optimal activity at neutral to mildly acidic pH (5.0–7.0) [1]. However, three Rha78s have been reported to display strongly acidic optimal pH 4.0–4.5 [22,24,25]. In contrast, fungal Rha78s typically prefer more acidic conditions (pH 4.0–6.0) [1]. Regarding thermal adaptation, most Rha78s show optimal activity at moderate temperatures (40~70 °C) [1], classifying them as mesophilic enzymes whose catalytic efficiency and thermostability often fall short of industrial requirements. Although a few thermophilic Rha78s have demonstrated promising thermostability [19,22,26], the Rha78s suitable for industrial biocatalytic applications remains limited. Therefore, the discovery of high-active and thermophilic α-L-rhamnosidases is of significant importance for advancing industrial processes under harsh conditions.

Rha78s typically employ a glutamic acid (Glu) or aspartic acid (Asp) as the catalytic general acid and a conserved Glu as the catalytic general base, hydrolyzing glycosidic bonds via general acid-base assisted inverting mechanism (single displacement) [27]. For instance, BtRha78A from *Bacteroides thetaiotaomicron* VPI-5482 achieves efficient hydrolysis through the synergistic action of Asp335 (general acid) and Glu595 (general base) [28]. Furthermore, the general acid motif from Asp330 to Asp342 provides insights into dynamic regulatory mechanisms among domains. At present, there are eight crystal structures of Rha78s, including BsRhaB from *Bacillus* sp. GL1 [27], BtRha78A [29], DtRha from *D. thermophilum* H-6-12 [18], KoRha from *Klebsiella michiganensis* KCTC 1686 [30], SaRha78A from *Streptomyces avermitilis* MA-4680 [31], AnRha from *Aspergillus niger* TS528 [29], AoRhaA from *Aspergillus oryzae* RIB40 [32], and AtRha from *Aspergillus terreus* CCF 3059 [33]. Structural analysis reveals that the α-L-rhamnosidases Rha78s possess characteristic domain architectures including one catalytic (α/α)_6_-barrel domain and several β-sheet domains [34].

Herein, we identified a novel Rha78 TsRha from *Thermotoga* sp. 2812B through genome mining. Bioinformatics tools were employed to analyze sequence alignment and phylogenetic relationships between TsRha and reported bacterial Rha78s. The enzymatic properties of TsRha were systematically characterized. Furthermore, homology modeling and molecular docking were performed to elucidate its domain architecture and the molecular basis of substrate selectivity. Finally, TsRha was applied in the biotransformation of a series of natural flavonoid glycosides to evaluate its conversion efficiency and potential for the biocatalytic preparation of rare and high-value flavonoid glucosides.

## 2. Materials and Methods

### 2.1. Bacterial Strains and Reagents

*E. coli* DH5α was used as the host for gene cloning. *E. coli* BL21(DE3) was used for protein over-expression. Flavonoid glycosides, including naringin (CAS: 10236-47-2, ≥95% purity), hesperidin (CAS: 520-26-3, ≥97% purity), naringin dihydrochalcone (CAS: 18916-17-1, ≥98% purity), rutin (CAS: 250249-75-3, ≥97% purity), troxerutin (CAS: 7085-55-4, ≥97% purity), and icariin (CAS: 489-32-7, ≥96% purity), were purchased from Aladdin (Shanghai, China). Neohesperidin (CAS: 13241-33-3, ≥95% purity), neohesperidin dihydrochalcone (CAS: 20702-77-6, 98% purity), and diosmin (CAS: 520-27-4, ≥95% purity) were provided by Yuanye (Shanghai, China). *p*-Nitrophenol (*p*NP) glycosides, including *p*NP-α-L-rhamnopyranoside (*p*NPαRha), *p*NP-α-D-glucopyranoside (*p*NPαGlc), *p*NP-α-D-galactopyranoside (*p*NPαGal), *p*NP-α-D-mannopyranoside (*p*NPαMan), *p*NP-β-D-glucopyranoside (*p*NPβGlc), *p*NP-β-D-galactopyranoside (*p*NPβGal), and *p*NP-β-D-xylopyranoside (*p*NPβXyl), were obtained from Aladdin (Shanghai, China). Chromatographic-grade acetonitrile and methanol were purchased from Merck (Darmstadt, Germany). All other chemicals for buffer and medium preparation were of analytical grade or higher.

### 2.2. Bioinformatic and Structural Analysis

The amino acid sequence of α-L-rhamnosidase TsRha from *Thermotoga* sp. 2812B and those of reported bacterial Rha78s were retrieved from the CAZy database [17]. Multiple sequence alignment was performed using ClustalW 2.0 [35] and visualized with ESPript 3.0 [36]. Phylogenetic tree was constructed via the neighbor-joining method using MEGA 12 [37]. The three-dimensional structure of TsRha was predicted using AlphaFold 2.3.2 available on the WeMol cloud platform [38]. Molecular docking of TsRha with naringin, rutin, and icariin was conducted using AutoDock-GPU v2 by WeMol cloud [38]. Structural visualization and analysis were performed using PyMOL educational version 3.1.4.1 [39].

### 2.3. Gene Cloning, Protein Over-Expression and Purification

The gene encoding α-L-rhamnosidase TsRha (GenBank accession: AIY87265) from *Thermotoga* sp. 2812b codon-optimized for *E. coli* using JCat, synthesized, and cloned into the pET-28a vector via *Nde* I and *Xho* I restriction sites with dual N- and C-terminal (His)_6_-tags by Sangon Biotech (Shanghai, China). *E. coli* BL21(DE3) harboring the recombinant plasmid pET-28a-*TsRha* was cultured in 2 × YT medium at 37 °C with shaking at 180 rpm until the OD_600_ reached 1.0. Protein expression was induced with 0.5 mM isopropyl-β-D-thiogalactopyranoside (IPTG) at 16 °C for 12 h. Cells were harvested by centrifugation (7000× *g*, 4 °C), resuspended in 50 mM NaH_2_PO_4_-Na_2_HPO_4_ buffer (PB, pH 7.0) at a ratio of 1:12 (*w*:*v*), and disrupted by ultrasonication. The cell lysate was centrifuged at 9000× *g* for 30 min at 4 °C, and the supernatant was heat-treated at 70 °C for 30 min. TsRha was purified by Ni-NTA affinity chromatography and dialyzed twice against 50 mM PB buffer (pH 7.0) containing 200 mM arginine before storage at −20 °C. Protein concentration was determined by measuring the absorbance at 280 nm (A_280_) using a NanoDrop2000 spectrophotometer (Thermo Fisher Scientific, Waltham, MA, USA). Protein purity was assessed by 12% sodium dodecyl sulfate-polyacrylamide gel electrophoresis (SDS-PAGE).

### 2.4. Enzymatic Characterization of TsRha

#### 2.4.1. Enzyme Activity Assay

The enzymatic properties of TsRha were characterized using naringin as the substrate. Standard reaction (200 μL) contained 180 μL of 50 mM citric acid-sodium citrate (CA) buffer (pH 5.0), 10 μL of 20 mM naringin (dissolved in DMSO), and 10 μL of TsRha (final concentration 0.05 mg mL^−1^). The mixture was incubated at 90 °C for 10 min with shaking at 800 rpm and terminated by adding 800 μL of methanol. After centrifugation (13,000 rpm, 5 min), the supernatant was filtered through a 0.22 μm nylon membrane for HPLC analysis. All enzyme assays were performed in three independent experiments. Enzymatic activity was determined by quantifying substrate concentrations via HPLC based on a standard curve. Enzymatic activity was calculated as follows:

Enzymatic activity (U mg^−1^) = (1 − [S])/(t × c) (1)

where [S] represents the residual substrate concentration (mM), t is the reaction time (5 min), and c is the final enzyme concentration (mg mL^−1^). One unit of enzyme activity (U) was defined as the amount of enzyme required to hydrolyze 1 μmol of substrate per minute at 90 °C and pH 5.0.

#### 2.4.2. Optimum pH and Temperature

The optimal pH of TsRha was determined by measuring activity at 80 °C in 50 mM buffers across pH 4.0–9.0, including CA buffer (pH 4.0–6.0), PB buffer (pH 6.0–8.0), and glycine-NaOH buffer (pH 8.0–9.0). The optimal temperature was assessed by measuring activity at temperatures ranging from 30 to 90 °C in 50 mM CA buffer (pH 5.0).

#### 2.4.3. Thermostability and Organic Solvent Tolerance

Thermal stability was evaluated by incubating TsRha (1.0 mg mL^−1^) at 80 °C or 90 °C for various durations with an additional layer of 200 μL paraffin oil to prevent evaporation, followed by measuring residual activity under standard reaction conditions. The effects of organic solvents (methanol, ethanol, ethylene glycol, isopropanol, dimethyl sulfoxide (DMSO), and glycerol) on enzymatic activity were explored by adding 10% (*v*/*v*) of each solvent to the reaction mixture. The reaction replacing organic solvent with water was used as the control.

#### 2.4.4. Substrate Selectivity

Catalytic activities of TsRha toward various natural flavonoid glycosides naringin (1), hesperidin (2), neohesperidin (3), naringin dihydrochalcone (4), neohesperidin dihydrochalcone (5), rutin (6), troxerutin (7), icariin (8), and diosmin (9) were determined under standard reaction conditions.

For *p*NP glycosides, reactions (200 μL) contained 170 μL of 50 mM CA buffer (pH 5.0), 20 μL of *p*NP glycosides (10 mM), and 10 μL of TsRha (final concentration 0.001 mg mL^−1^ for *p*NPαRha and 0.2 mg mL^−1^ for other *p*NP glycosides). Reactions were performed at 90 °C for 10 min and terminated by adding 200 μL of 1.0 mM Na_2_CO_3_ solution. The mixture was diluted with 600 µL ddH_2_O, and absorbance at 405 nm was measured using a UV-5800PC spectrophotometer (METASH, Shanghai, China). All assays were performed in triplicate. Enzymatic activity was calculated as:

Enzymatic activity (U mg^−1^) = (ΔA_405_ × 5)/(t × ε × l × c) × 1000 (2)

where ΔA_405_ is the change in absorbance at 405 nm; t is the reaction time (10 min), c is the final enzyme concentration (mg mL^−1^), ε is the molar extinction coefficient of *p*NP (16,000 L mol^−1^ cm^−1^), and l is the path length (1 cm). One unit of activity (U) was defined as the amount of enzyme required to release 1 μmol of *p*NP per minute at 90 °C and pH 5.0.

### 2.5. Biotransformation of Natural Flavonoid Glycosides by TsRha

Biotransformation of naringin, hesperidin, neohesperidin dihydrochalcone, rutin, troxerutin, and icariin into the corresponding glucosides were performed using TsRha in the standard reaction system for varying durations. All experiments were conducted in triplicate. Conversion rate was calculated as follows:

Conversion rate (%) = (C_0_ − C_1_)/C_0_ × 100% (3)

where C_0_ represents the initial substrate concentration (1.0 mM), and C_1_ indicates the residual substrate concentration.

### 2.6. High-Performance Liquid Chromatography (HPLC)

HPLC analysis was performed using a Waters 1525 binary pump and a Waters 2487 dual λ absorbance detector (Waters, Framingham, MA, USA) equipped with a reverse-phase Hypersil OSD2-C18 column (4.6 × 150 mm, particle size 5 µm, Elite, Dalian, China) at room temperature. Substrates and de-rhamnosylated products were separated using mobile phase consisting of 0.5% (*v*/*v*) acetic acid (A) and acetonitrile (B) in varying ratios (Table S1) at a flow rate of 1.0 mL min^−1^ and detected at specific wavelengths (Table S1).

## 3. Results

### 3.1. Bioinformatics and Structural Analysis for TsRha

**Figure 1:**
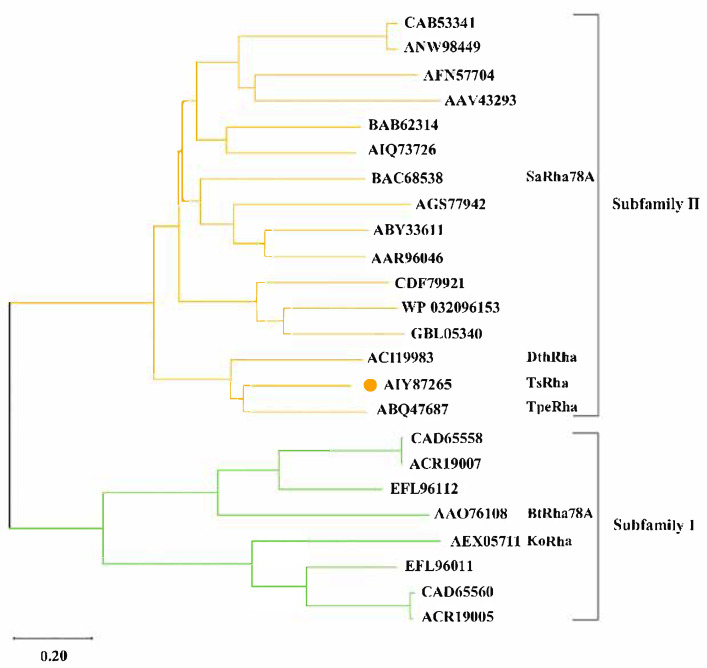
Phylogenetic tree and subfamily classification of TsRha. Phylogenetic tree for TsRha and 23 bacterial Rha78s was constructed via the neighbor-joining method using MEGA 12 [37]. TsRha was labeled by an orange circle.

**Figure 2:**
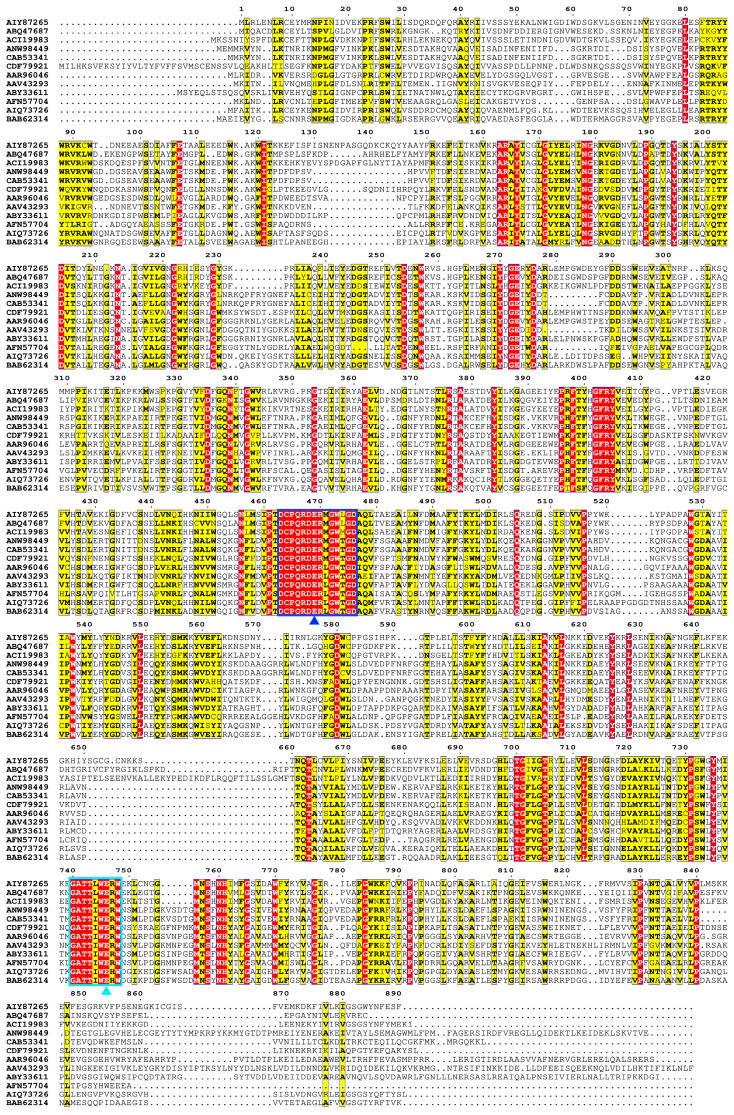
Multiple sequence alignment of TsRha with homologous bacterial Rha78s. Multiple sequence alignment was performed using ClustalW 2.0 [35] and visualized with ESPript 3.0 [36]. Residues marked with blue and cyan triangles represent the general acid and general base, respectively. Regions enclosed by blue and cyan rectangles represent general acid motif and general base motif, respectively. Residues shaded in red and yellow represent completely conserved and highly conserved residues, respectively.

The α-L-rhamnosidase TsRha gene comprises 2673 bp and encodes a protein of 890 amnio acid residues. Based on the difference in the catalytic general acid, Rha78s are classified into two subfamilies: subfamily I utilizes Asp as the general acid, whereas subfamily II employs Glu [27]. Phylogenetic analysis revealed that TsRha clusters within subfamily II and forms a distinct clade with two bacterial Rha78s, indicating a close evolutionary relationship and high sequence homology with TpeRha and DtRha (Figure 1). Sequence alignment of TsRha with homologous bacterial Rhs78s suggested that Glu469 and Glu746 in TsRha serve as the general acid and general base, respectively. The general acid motif (DCPQRDERMGWLGD, residues 463–476) and general base motif (GATTLWERW, residues 740–748) are highly conserved (Figure 2).

**Figure 3:**
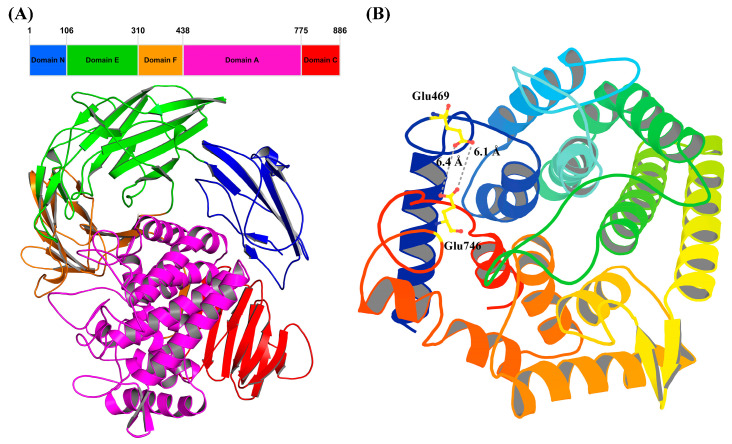
Domain architecture (**A**) and catalytic domain (**B**) of TsRha. Structural visualization and analysis were performed using PyMOL educational version 3.1.4.1 [39]. The catalytic general acid and base Glu469 and Glu746 are displayed by yellow ball-and-stick models. The distances between Glu469 and Glu746 were indicated by grey dashed lines.

The predicted 3D structure of TsRha consists of five distinct domains (Figure 3A), including one α-helical domain catalytic domain A (residues 439–775) and four β-sheet domains, domain N (residues 1–106), domain E (residues 107–310), domain F (residues 311–438), and domain C (residues 776–886). Domain A constitutes catalytic domain, featuring the characteristic (α/α)_6_ barrel typical of Rha78s. Different Rha78s possess distinct (1–5) β-sheet domains. TsRha exhibits a domain architecture similar to those of DtRha [18] and AtRha [33]. TsRha likely catalyzes glycosidic bonds via an inverting catalysis mechanism. The distances between Glu469 and Glu746 in TsRha were 6.1 Å and 6.4 Å (Figure 3B), within the typical range for inverting glycoside hydrolases.

### 3.2. Heterologous Expression and Purification of TsRha

**Figure 4:**
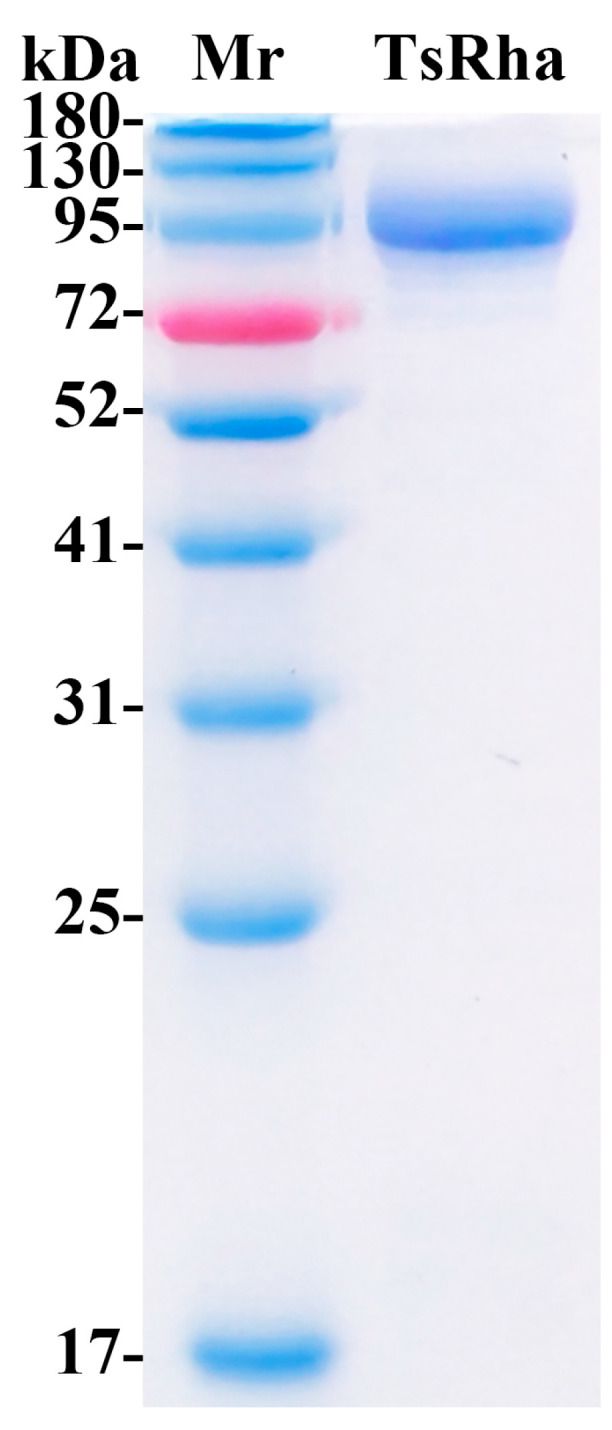
SDS-PAGE for purified TsRha. (Original image in the Supplementary Materials Figure S2). The theoretical molecular weight of recombinant TsRha with dual (His)_6_-tags is 106.4 kDa.

TsRha was successfully overexpressed in *E. coli* BL21(DE3). Following Ni-NTA affinity purification, SDS-PAGE analysis revealed a single band corresponding to the theoretical molecular weight (Figure 4), indicating high purity.

### 3.3. Enzymatic Properties of Recombinant TsRha

#### 3.3.1. Optimal pH and Temperature

**Figure 5:**
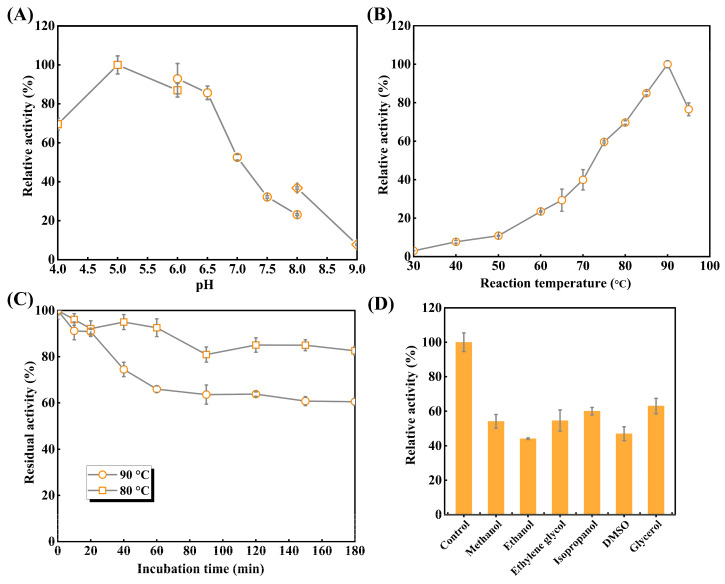
Enzymatic properties of recombinant TsRha. (**A**) Optimal pH of TsRha. 50 mM citric acid-sodium citrate buffer (pH 4.0–6.0, square), NaH_2_PO_4_–Na_2_HPO_4_ buffer (pH 6.0–8.0, circle), and glycine–NaOH buffer (pH 8.0–9.0, diamond) were used. Enzyme activity in 50 mM CA buffer (pH 5.0) is defined as 100%. (**B**) Optimal temperature of TsRha. Enzyme activity at 90 °C is defined as 100%. (**C**) Thermal inactivation curves of TsRha. Enzyme activity without the incubation is defined as 100%. (**D**) Organic solvent tolerance of TsRha. Enzyme activity replacing organic solvent with ddH_2_O used as the control is defined as 100%. All enzyme assays were performed in three independent experiments. Data are presented as mean ± standard deviation.

As shown in Figure 5A, TsRha exhibited maximum activity at pH 5.0. The enzyme retained >85.7% of its activity within the acidic pH range of 5.0–6.5 and maintained 69.6% activity even in pH 4.0, indicating its acidophilic nature. However, activity declined sharply above pH 7.5, retaining only 7.7% at pH 9.0.

The effects of temperatures on catalytic activities of TsRha were explored (Figure 5B). Catalytic activity of TsRha increased progressively from 30 to 90 °C, reaching a maximum at 90 °C. TsRha retained 76.6% of enzymatic activity even at 95 °C but showed lower activity (<39.9%) at temperatures between 30 and 70 °C. The high hydrolytic activity (≥59.6%) observed at 75–95 °C reflects the thermophilic character of TsRha.

#### 3.3.2. Thermal Stability and Organic Solvent Tolerance

Thermal inactivation curves (Figure 5C) demonstrated that TsRha retained 92.5% of residual activity after 60 min at 80 °C, and 82.6% of residual activity after 180 min. At 90 °C, 90.8% of catalytic activity was residual after 20 min. Beyond 60 min, no further notable decline in catalytic activity was observed, and 60.5% of catalytic activity was preserved even after 180 min, indicating favorable thermostability at 80 °C and 90 °C.

Organic solvent tolerance is crucial for industrial applications; DMSO and methanol are often applied to enhance the solubility of flavonoid glycosides. TsRha maintained 44.1~63.0% of catalytic activity in the presence of 10% (*v*/*v*) of organic solvents (Figure 5D), demonstrating considerable tolerance to organic solvent.

#### 3.3.3. Substrate Selectivity

Substrate selectivity was evaluated using both *p*NP-glycosides and natural flavonoid glycosides. For *p*NP-glycosides (Table 1), TsRha showed the highest activity toward *p*NPαRha (31.7 U mg^−1^). Weak activities were detected for *p*NPβGlc (0.04 U mg^−1^) and *p*NPβGal (0.02 U mg^−1^). It was suggested that TsRha exhibited weak catalytic capability of β-D-glucosidase and β-D-galcosidase. No activity was observed toward *p*NPαGlc, *p*NPαGal, *p*NPαMan, or *p*NPβXyl.

**Figure 6:**
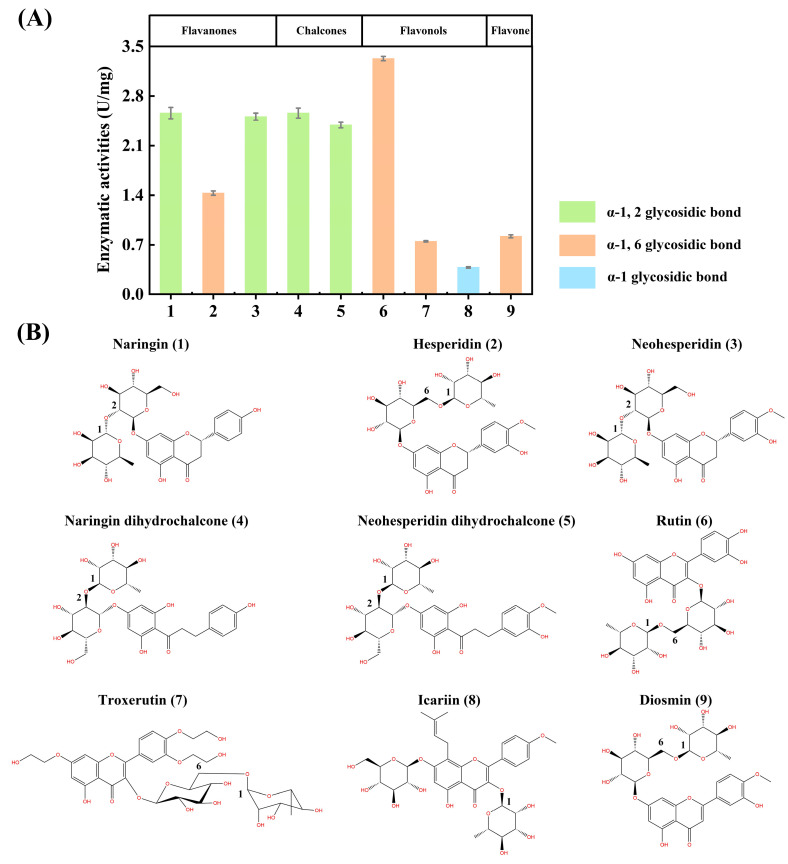
Substrate selectivity of TsRha on natural flavonoid glycosides. (**A**) Enzymatic activities on natural flavonoid glycosides with various glycosidic bonds from different flavonoid subgroups. All assays were performed in three independent experiments. Data are presented as mean ± standard deviation. (**B**) Molecular structures of the natural flavonoid glycosides tested.

TsRha displayed considerable activity (2.4~2.6 U/mg) toward flavanone diglycosides (1 and 3) and dihydrochalcone diglycosides (4 and 5) containing α-1, 2 glycosidic linkages, compared to hesperidin (2) which possesses an α-1, 6 glycosidic bond (Figure 6). The highest activity among natural flavonoid glycosides was observed for the flavonol diglycoside rutin (6, 3.3 U/mg), which also contains an α-1, 6 linkages (Figure 6). Moderate activity (0.8 U/mg) was detected toward the flavone diglycoside diosmin (9, α-1, 6 linkage). TsRha also catalyzed the hydrolysis of troxerutin (7, 0.8 U/mg) with the modification of ethylene glycol and an α-1, 6 glycosidic bond (Figure 6). Additionally, a certain level of activity (0.4 U/mg) was observed toward the prenylated flavonoid glycoside icariin (8) containing an α-1 glycosidic bond (Figure 6). These results indicated that TsRha possesses a broad substrate spectrum, accepting natural flavonoid glycosides with different glycosidic bonds (α-1, 2, α-1, 6, α-1) from diverse flavonoid subgroups (flavanone, flavone, flavonol, and dihydrochalcone) (Figure 6).

### 3.4. Biotransformation of Natural Flavonoid Glycosides by TsRha

**Figure 7:**
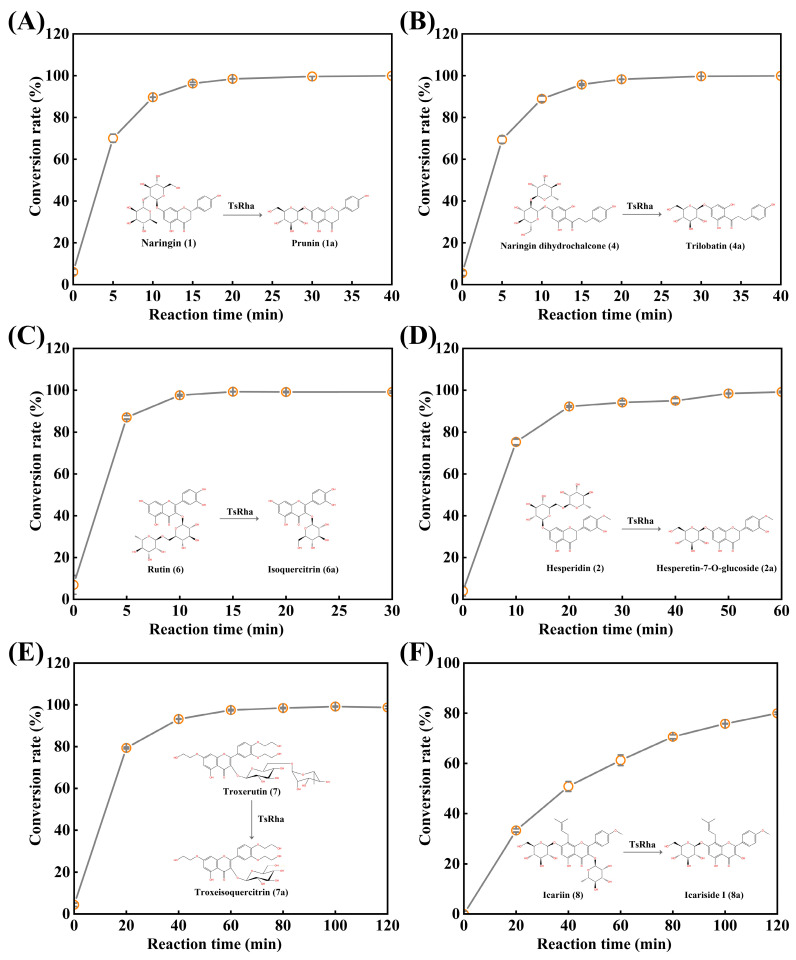
Biotransformation of natural flavonoid glycosides into rare flavonoid glucosides by TsRha. (**A**) Reaction time curve for biotransformation of naringin into prunin. (**B**) Reaction time curve for biotransformation of naringin dihydrochalcone into trilobatin. (**C**) Reaction time curve for biotransformation of rutin into isoquercitrin. (**D**) Reaction time curve for biotransformation of hesperidin into hesperetin-7-O-glucoside. (**E**) Reaction time curve for biotransformation of troxerutin into troxeisoqueritrin. (**F**) Reaction time curve for biotransformation of icariin into icariside I. Conversion pathways of natural flavonoid glycosides into the corresponding flavonoid glucosides are presented. All assays were performed in three independent experiments. Data are presented as mean ± standard deviation.

Given its broad substrate selectivity, the biotransformation potential of TsRha was evaluated for representative flavonoid glycosides (Figure 7). For the conversion of naringin to prunin (Figure 7A), the conversion rate increased rapidly to 96.3% within 15 min, reaching a plateau at 20 min with a final conversion of 99.9%. Similarly, naringin dihydrochalcone was rapidly hydrolyzed to trilobatin (95.8% in 15 min), achieving a maximum conversion of 99.9% at 40 min (Figure 7B).

Rutin was almost completely converted to isoquercitrin (97.6%) within 10 min, with the conversion rate reaching 99.3% by 15 min (Figure 7C). For the conversion of hesperidin to hesperetin-7-O-glucoside (Figure 7D), the conversion rate increased rapidly within 20 min, reaching 99.1% at 60 min. In the biotransformation of troxerutin (Figure 7E), conversion progressed gradually, attaining 99.2% at 100 min. Finally, TsRha catalyzed the hydrolysis of icariin to rare icariside I, achieving a conversion rate of 80.0% at 120 min (Figure 7F).

## 4. Discussion

The α-L-rhamnosidase TsRha exhibited optimal activity at pH 5.0, classifying it as an acidophilic enzyme, whereas most bacterial Rha78s display neutral or slightly acidic pH optima [1]. Its optimum temperature of 90 °C is higher than those of most reported Rha78s (40–70 °C) [1] and is comparable to those of TpeRha (90 °C) [22] and DtRha (95 °C) [18]. Elevated reaction temperatures can improve substrate solubility and conversion efficiency in the biotransformation of natural flavonoid glycosides.

TsRha demonstrated notable thermostability, retaining 82.6% and 60.5% residual activities after 3 h at 80 °C and 90 °C. In contrast, most microbial Rha78s are stable only below 60 °C [1], and PdRha from *Parabacteroides distasonis* loses stability above 50 °C [40]. TsRha also outperformed the thermostable TpeRha, which retained only ~45% activity after 2 h at 80 °C and was inactive at 90 °C [22]. Thermophilic enzymes often evolve unique structural adaptations to maintain stability and functionality at high temperatures [41]. Organic solvents are frequently employed to dissolve hydrophobic substrates in the biotransformation. TsRha maintained a certain level of functionality (44.1~63.0%) in 10% (*v*/*v*) organic solvents. This is lower compared to TpeRha [22] and St-Rha [13] in 10% (*v*/*v*) organic solvents, whereas BtRha78A showed tolerance only at 5% (*v*/*v*) [28]. This property suggests its suitability for reaction systems containing certain concentrations of organic solvents, including organic–aqueous biphasic systems, which can enhance substrate solubility and reaction efficiency [42,43].

**Figure 8:**
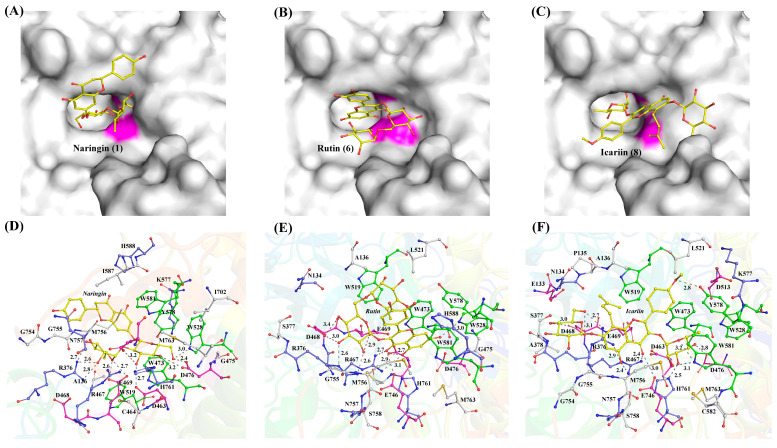
Binding modes and interactions of naringin (**A**,**D**), rutin (**B**,**E**), and icariin (**C**,**F**) with the residues in the substrate-binding pocket of TsRha. The general acid and general base are shown in magenta in (**A**–**C**). Flavonoid glycosides are depicted as yellow ball-and-stick models. Acidic, basic, and aromatic residues are presented in magenta, blue, and green ball-and-stick models, respectively. Hydrogen bonds are indicated by grey dashed lines.

Substrate selectivity among α-L-rhamnosidases towards natural flavonoid glycosides varies considerably with the types of glycosidic bonds and the positions of glycosylation linked to the aglycones. Understanding these preference is crucial for selecting appropriate enzymes to produce high-value flavonoid glucosides. TsRha displayed a broad substrate spectrum on natural flavonoid glycosides, showing high activities on natural flavonoid diglycosides with α-1, 2 and α-1, 6 glycosidic bonds from flavanones, dihydrochalcones, flavonols and flavones, as well as moderate activity toward icariin (α-1 glycosidic bond). Molecular docking revealed that naringin, rutin, and icariin bind to the substrate-binding pocket of TsRha with a reasonable conformation forming hydrogen bonds and π-π stacking interactions with aromatic residues (Figure 8).

While most α-L-rhamnosidases hydrolyze α-1, 2 and/or α-1, 6 glycosidic bonds [1,44], few were active toward α-1 glycosidic bond [1]. DtRha hydrolyzes flavonoid diglycosides with α-1, 6 and α-1, 2 glycosidic linkages [18], whereas BtRha78A is specific for α-1, 6 glycosidic bond [44]. Human gut bacterial α-L-rhamnosidases HFM-RhaC also acts on α-1, 6 and α-1, 2 glycosidic bonds [44]. However, BtRha78A and HFM-RhaC were inactive toward α-1 glycosidic bond [44]. Medicinal plants such as *Citrus*, *Lonicera japonica*, and *Chrysanthemum* contain diverse natural flavonoid glycosides with varied sugar moieties, glycosidic bonds, and aglycone subgroups. The broad substrate specificity of TsRha enables the hydrolysis of complex natural flavonoid extracts into flavonoid glucosides and/or aglycones, potentially enhancing the pharmacological efficacy.

De-rhamnosylation of natural flavonoid diglycosides can significantly improve water solubility, bioavailability, and bioactivity. TsRha demonstrated high efficiency in converting naringin, naringin dihydrochalcone, rutin, hesperidin and troxerutin into their corresponding glucosides prunin, trilobatin, isoquercitrin, hesperetin-7-O-glucoside, and troxeisoquercitrin, respectively. Using only 0.05 mg mL^−1^ enzyme, near-quantitative conversion (≥99.1%) of 1 mM natural flavonoid diglycosides was achieved within 20~100 min. Although conversion of icariin was moderate (80% at 120 min), TsRha is among the few α-L-rhamnosidases capable of hydrolyzing the α-L-rhamnosyl moiety of icariin to produce rare icariside I, offering a novel alternative for the biotransformation of icariin.

## 5. Conclusions

In summary, a novel GH78 α-L-rhamnosidase TsRha was identified through genome mining and comprehensively characterized via bioinformatics, computer-aided structural analysis, and biochemical characterization. Sequence alignment indicated that Glu469 and Glu746 in TsRha serve as the catalytic general acid and general base, respectively. TsRha possesses a domain architecture comprising four β-sheet domains and one catalytic (α/α)_6_-barrel domain. Enzymatic characterization revealed optimal activity at 90 °C and pH 5.0, remarkable thermostability at 80 °C, and considerable tolerance to organic solvents. TsRha exhibited broad substrate selectivity toward various natural flavonoid glycosides with different glycosidic bonds from diverse aglycone subgroups. Furthermore, it demonstrated high conversion efficiencies toward a variety of natural flavonoid diglycosides, achieving ≥99.1% conversion within 20~100 min. The excellent properties of TsRha including high activity, acidophilicity, thermophilicity, good thermostability and broad substrate spectrum make it a powerful biocatalytic tool for the efficient production of rare and high-value flavonoid glucosides with improved bioavailability and bioactivity from natural flavonoid diglycosides.

## Figures and Tables

**Table 1:** Enzymatic activities of TsRha on various *p*NP glycosides.

*p*NP Glycosides	Enzymatic Activities (U/mg)
*p*NPαRha	31.7 ± 1.0
*p*NPαGlc	NA
*p*NPαGal	NA
*p*NPαMan	NA
*p*NPβGlc	3.5 × 10^−2^ ± 0.1 × 10^−2^
*p*NPβGal	2.1 × 10^−2^ ± 0.3 × 10^−2^
*p*NPβXyl	NA

## Data Availability

The original contributions presented in this study are included in the article. Further inquiries can be directed to the corresponding authors.

## Supplementary Materials

The following supporting information can be downloaded at https://www.mdpi.com/article/10.3390/biom16010068/s1, Table S1: HPLC conditions for separation and detection of the substrates flavonoid glycosides and the corresponding deglycosylated products; Figure S1: Map of the recombinant plasmid pET-28a-TsRha; Figure S2: Original image of SDS-PAGE in Figure 4; Figure S3: Standard curves of the substrates natural flavonoids glycosides for HPLC.

## Abbreviations

The following abbreviations are used in this manuscript:

CA	Citric acid-sodium citrate
PB	NaH2PO4-Na2HPO4
DMSO	Dimethyl sulfoxide
pNP	p-Nitrophenol
GH	Glycoside hydrolase
Rha78	GH78 α-L-rhamnosidase
IPTG	Isopropyl-β-D-thiogalactoside
HPLC	High-performance liquid chromatography
