# Supplementary material for: Characterization of a Thermophilic and Acidophilic GH78 α-L-Rhamnosidase from Thermotoga sp. 2812B Capable of Efficiently Hydrolyzing a Variety of Natural Flavonoid Diglycosides
Source: Biomolecules. 2025 Dec 31;16(1):68. doi: 10.3390/biom16010068 (PMC12838782; doi:10.3390/biom16010068)
Supplement: Supplementary file 1 [file biomolecules-16-00068-s001.zip › biomolecules-4048162-supplementary.pdf]

Table S1. HPLC conditions for separation and detection of the substrates flavonoid glycosides and the corresponding deglycosylated products.

Substrates	Products	Detection wavelength (nm)	Mobile phase 0.5% (v/v) acetic acid (A) : acetonitrile (B)
Naringin (1)	Prunin	283	77:23
Hesperidin (2)	Hesperetin-7-O-glucoside	283	77:23
Neohesperidin (3)	Hesperetin-7-O-glucoside	283	77:23
Naringin dihydrochalcone (4)	Trilobatin	283	74:26
Neohesperidin dihydrochalcone (5)	Hesperetin-7-O-glucoside dihydrochalcone	283	74:26
Rutin (6)	Isoquercitrin	260	84:16
Troxerutin (7)	Troxeisoquercitrin	260	82:18
Icariin (8)	Icariside Ⅰ	270	64:36
Diosmin (9)	Diosmetin-7-O-glucoside	340	77:23

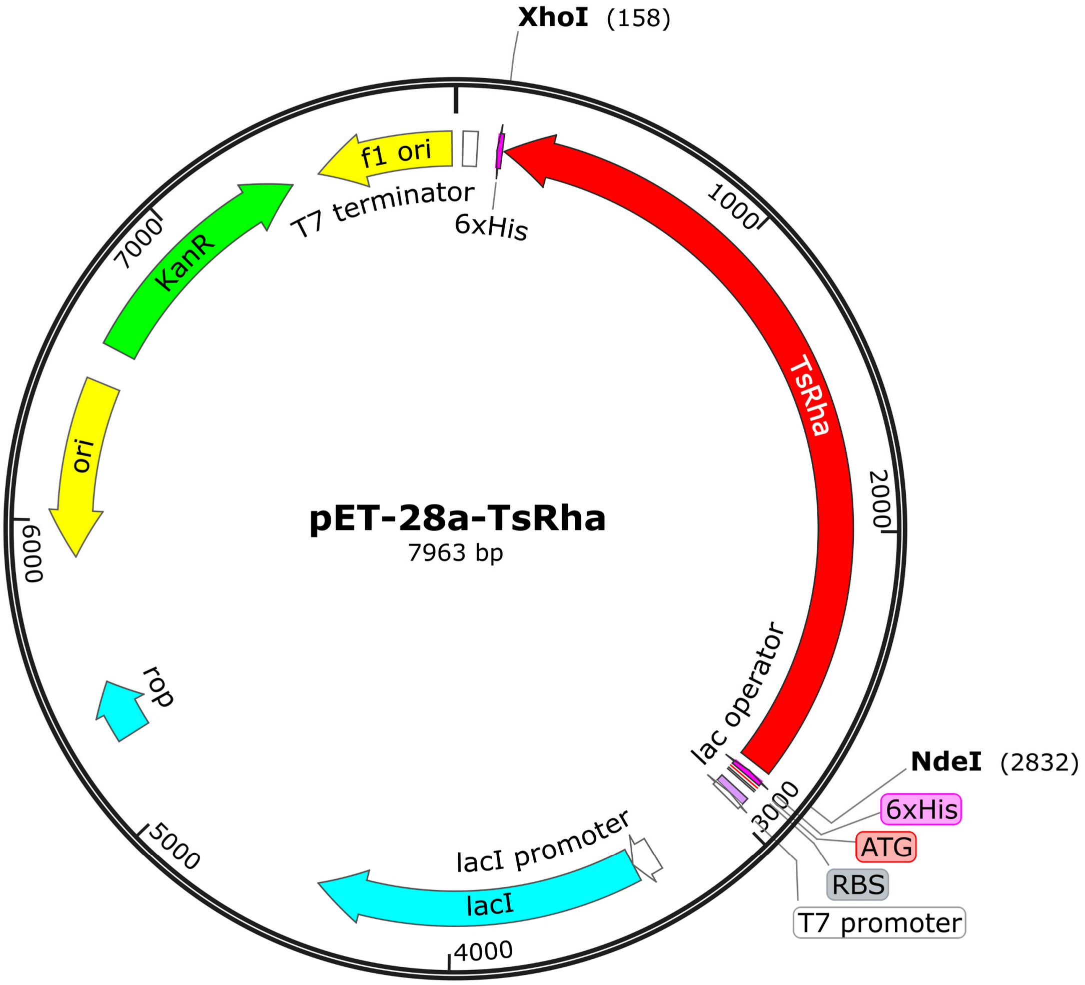

Figure S1. Map of the recombinant plasmid pET-28a-*TsRha*.

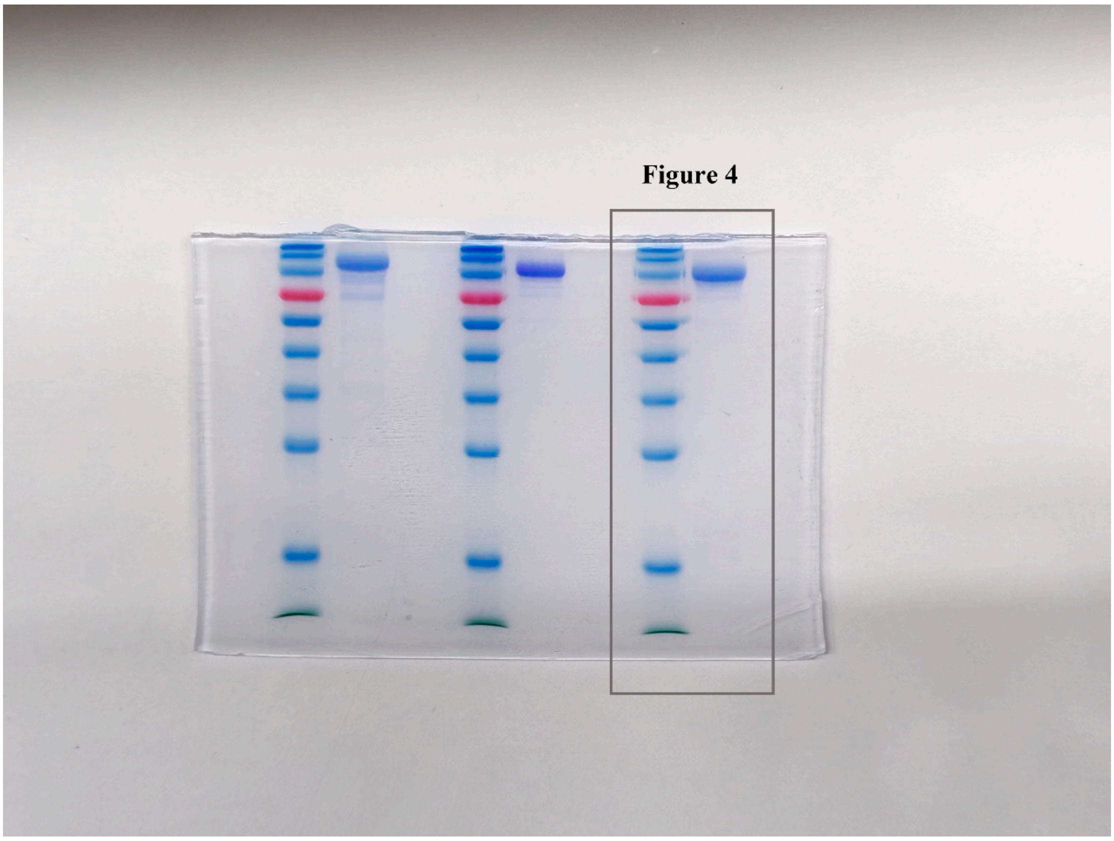

Figure S2. Original image of SDS-PAGE in Figure 4.

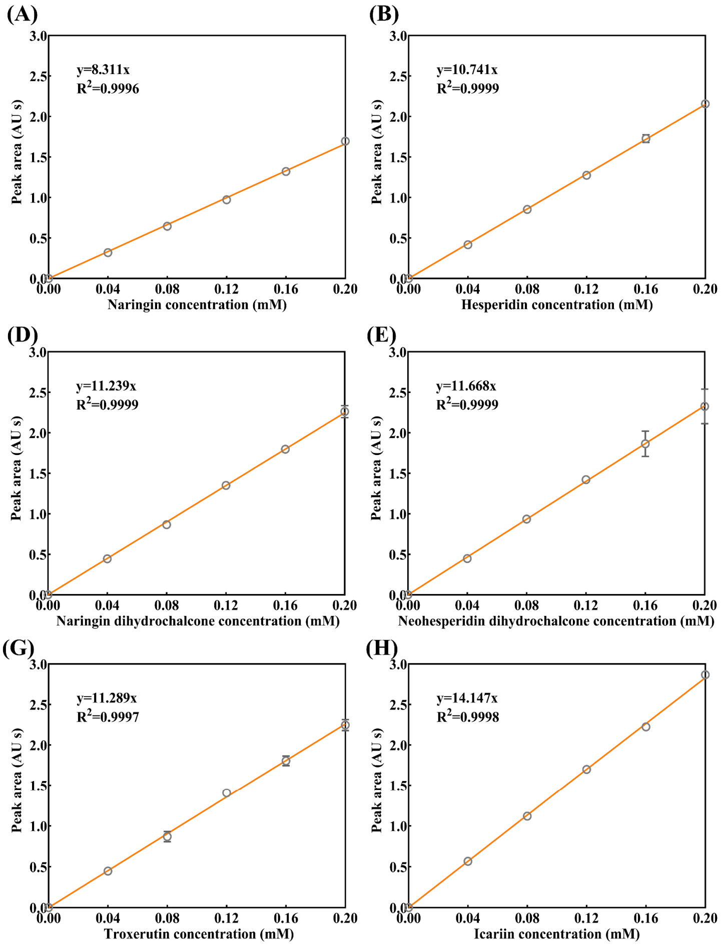

Figure S3. Standard curves of the substrates natural flavonoids glycosides for HPLC. All enzyme assays were performed in three independent experiments. Data are presented as mean ± standard deviation.
